# Type and antibiotic susceptibility of bacteria cultured in paediatric acute appendicitis

**DOI:** 10.4102/sajid.v40i1.689

**Published:** 2025-02-28

**Authors:** Elizabeth Brits, Estie Kruger, Karlize Fivaz, Koot Oosthuizen, Mariska Joubert, Petro-Mari van Pletzen, Ronelle Roux, Tahlita Fourie, Trewhella van Aswegen, Joseph B. Sempa, Susanna le Grange

**Affiliations:** 1 Department of Surgery, Faculty of Health Sciences, University of the Free State, Bloemfontein, South Africa; 2 Department of Biostatistics, Faculty of Health Sciences, University of the Free State, Bloemfontein, South Africa

**Keywords:** acute appendicitis, microbe, antibiotic sensitivity, paediatric, resistance

## Abstract

**Background:**

Studying the microbial profile and their antibiotic resistance in paediatric appendicitis is essential for tracking susceptibility, guiding treatment choices and ensuring effectiveness. Understanding variations in therapies can improve outcomes and reduce complications. Despite its importance, limited research has been conducted in South Africa on microbial profiles and antibiotic resistance in paediatric appendicitis.

**Objectives:**

To identify bacteria cultured from pus specimens obtained from paediatric patients with acute appendicitis and determine their antibiotic susceptibility.

**Method:**

This was a prospective case series of children aged 13 years and younger, who had appendectomies for acute appendicitis. Data were collected via REDCap and analysed using R software. Pus swabs were obtained for microscopy, culture and sensitivity of organisms isolated.

**Results:**

The study comprised 20 patients, of whom 12 (60%) were male. Most cases (*n* = 17; 85%) were complicated appendicitis. *Escherichia coli* was the most prevalent bacterial species isolated, accounting for 60% of cases, while no bacterial growth was observed in 30% of cases. All the isolates (100%) were susceptible to cefepime, gentamicin, amikacin, ertapenem, imipenem and meropenem, while high sensitivity rates of 92.9% were found for ciprofloxacin, ceftazidime and piperacillin-tazobactam. Short-term complications (predominantly surgical site infections) were present in 6 patients (30%).

**Conclusion:**

*Escherichia coli* was the most common bacterium in paediatric acute appendicitis, with all isolates sensitive to ciprofloxacin.

**Contribution:**

Regional monitoring and research are useful to adapt protocols and combat increasing antibiotic resistance.

## Introduction

Acute appendicitis, the leading cause of emergency surgery worldwide, is a common and severe paediatric condition that requires urgent diagnosis and treatment to prevent severe complications such as perforation and peritonitis.^1,2,3,4^ Symptomatology includes abdominal pain, fever and vomiting,^5^ and the diagnosis is confirmed with imaging, mainly ultrasound and/or computed tomography.^5,6^ Treatment usually involves an appendectomy, either laparoscopic or open. Postoperative care focuses on pain management and minimising complications.^5,6^

The appendiceal microbiome plays a crucial role in the pathogenesis of acute appendicitis.^7,8^ Age, environment, diet and antibiotic use can disrupt this microbiome, increasing the risk of appendicitis.^9,10^ Recent studies suggested that microbial dysbiosis in the appendix may trigger inflammation, leading to acute appendicitis.^11,12,13,14^ Microbial shifts may lead to new therapies to restore a healthy microbiome and prevent appendicitis.^13,15^ Probiotics and dietary changes show promise to reduce the risk in vulnerable groups. Genetic research could improve tailored treatments. Understanding how these factors interact with microbes is key to developing prevention strategies targeting genetics and lifestyle. Integrating this research into public health efforts on gut health and its link to appendicitis is crucial.^13,15^

Identifying microorganisms cultured in association with acute appendicitis and their antibiotic sensitivity is an important component of effective treatment. Targeted antimicrobial therapy reduces complications and limits broad-spectrum antibiotic use.^16^ With increasing antibiotic resistance, region-specific data are essential, as empiric treatment can lead to ineffective therapies and higher costs.^17,18^ As non-operative antibiotic management gains popularity, understanding local resistance patterns becomes even more important.^3,19,20^ This approach enhances antibiotic stewardship and improves treatment outcomes.^1,18,21^

Limited information is available on the microorganisms cultured in acute appendicitis and their antibiotic susceptibility in the South African paediatric population, with no data available from our own unit.^22^ Given the increasing challenge of antibiotic resistance, region-specific research is vital. This study addresses this need by identifying the microorganisms isolated in acute appendicitis in children under 13 years of age in central South Africa, and evaluating the antibiotic susceptibility patterns of these organisms. The study was conducted from July 2023 to November 2023 aiming to provide data to inform more effective treatment strategies based on the most recent antibiotic susceptibility profiles, enhance patient care, and contribute to the global effort against antibiotic resistance.

## Methods

### Study sample and measurement

This prospective descriptive study involved 20 patients under 13 years of age who underwent an appendectomy for acute appendicitis at the Paediatric Surgical Department in Pelonomi Tertiary Hospital, Bloemfontein, from July 2023 to November 2023. The standard empiric treatment for acute appendicitis during the study period was amoxicillin-clavulanic acid adjusted to intraoperative pus swab microscopy, culture and sensitivity (MCS) results. Patients treated non-operatively and those with missing microbiology test results were excluded. All patients were referred from peripheral hospitals or clinics, and no data pertaining to initial antibiotics or stat treatment provided by the referral institutions were documented. A pus swab was taken during the appendectomy for MCS to determine the identified organisms and antimicrobial drug sensitivities. Agar plates were also incubated under anaerobic conditions for the isolation of potential anaerobes. No blood cultures were sent for analysis. All methods were performed according to the standard operating procedures (SOPs) of the local medical microbiology laboratory. Basic demographic information, appendicitis classification,^23^ and MCS results were captured using REDCap (REDCap Consortium; Nashville, TN, US), an electronic data capturing tool hosted by the University of the Free State. Appendicitis is uncomplicated when confined to the appendix without perforation, abscess formation, or phlegmon, managed with appendectomy or occasionally antibiotics. Complicated cases involve perforation, abscess formation, or phlegmon, requiring surgery and prolonged antibiotics.^23,24^

### Data analysis

The Department of Biostatistics, Faculty of Health Sciences, UFS analysed the data using the statistical analysis software R (The R Foundation; Vienna, Austria).^25^ The results were summarised as frequencies and percentages.

### Ethical considerations

Ethical approval was obtained from the Health Sciences Research Ethics Committee (HSREC) of the University of the Free State (reference no. UFS-HSD2023/0465/2908) and the Free State Province Department of Health. Informed consent was obtained from the parents and/or legal guardians and assent from patients old enough to understand. Patient information was anonymised, and no identifying details were documented. Data were stored on REDCap, which was password-protected.

## Results

### Study population

The study comprised 20 patients, of whom 12 (60%) were male. The ages ranged from 4 years to 12 years, with a mean of 8 years. All the surgical procedures were open appendectomies performed via a Lanz incision. Most patients had complicated appendicitis (*n* = 17; 85%). Short-term complications occurred in 6 patients (30%), including surgical site infection (*n* = 4), intra-abdominal pus collection (*n* = 1) and adhesive bowel obstruction (*n* = 1). There were no mortalities, and all patients were discharged from the hospital.

### Isolated bacterial species

Table 1 shows the number of isolated bacteria in males and females. *Escherichia coli* was the most prevalent (*n* = 12; 60%). No bacterial growth was observed in 30% (*n* = 6) of cases. An extended-spectrum β-lactamase (ESBL) producing isolate (*Enterobacter cloacae* complex) was cultured from the pus specimen of one patient (*n* = 1/20; 5%). Bacteria isolated in the complicated appendicitis cases were *E.coli* (*n* = 4), *Enterobacter cloacae* complex (*n* = 1), and *Pseudomonas aeruginosa* (*n* = 1); no growth was observed in four cases.

**TABLE 1:** Bacterial species isolated from paediatric patients with acute appendicitis (*N* = 20).

Species	Total (*N*)	Male (*N* = 12)		Female (*N* = 8)		Total (%)
		*n*	%	*n*	%	
*Escherichia coli*	12	6	50.0	6	75.0	60.0
*Pseudomonas aeruginosa*	1	1	8.3	0	0.0	5.0
*Enterobacter cloacae* complex	1	0	0.0	1	12.5	5.0
No growth	6	5	41.7	1	12.5	30.0

### Antibiotic susceptibility

Table 2 shows the antibiotic sensitivity and resistance of the isolated bacteria (*n* = 14). All isolates (*n* = 14; 100%) were susceptible to cefepime, amikacin, imipenem and meropenem, followed by a 92.9% (*n* = 13) sensitivity rate to ciprofloxacin, ceftazidime, gentamicin, piperacillin-tazobactam, ertapenem, colistin and tigecycline.

**TABLE 2:** Antibiotic susceptibility and resistance of all bacteria isolated from acute appendicitis pus swabs against the drugs listed (*N* = 14).

Antimicrobial agent	Profile of isolates cultured						
	Susceptible		Resistant		Intermediate		Not tested (*n*)
	*n*	%	*n*	%	*n*	%	
Trimethoprim-sulfamethoxazole	9	64.3	5	35.7	0	0.0	-
Ampicillin and/or amoxicillin	7	53.8	6	46.2	0	0.0	1
Amoxicillin-clavulanic acid	10	76.9	3	23.1	0	0.0	1
Nitrofurantoin	12	85.7	1	7.1	1	7.1	-
Ciprofloxacin	13	92.9	1	7.1	0	0.0	-
Cefuroxime (parenteral)	12	85.7	2	14.3	0	0.0	-
Cefuroxime (oral)	11	78.6	2	14.3	1	7.1	-
Cefoxitin	12	85.7	2	14.3	0	0.0	-
Cefotaxime and/or ceftriaxone	12	85.7	2	14.3	0	0.0	-
Ceftazidime	13	92.9	1	7.1	0	0.0	-
Cefepime	14	100.0	0	0.0	0	0.0	-
Gentamicin	13	100.0	0	0.0	0	0.0	1
Amikacin	14	100.0	0	0.0	0	0.0	-
Piperacillin-tazobactam	13	92.9	1	7.1	0	0.0	-
Ertapenem	13	100.0	0	0.0	0	0.0	1
Imipenem	14	100.0	0	0.0	0	0.0	-
Meropenem	14	100.0	0	0.0	0	0.0	-
Tigecycline	13	92.9	1	7.1	0	0.0	-

Note: The susceptibility profile for specific bacterial isolates is shown in Table 3. Of the 12 *Escherichia coli* isolates, 5 (41.7%) were resistant to ampicillin and/or amoxicillin, followed by trimethoprim-sulfamethoxazole (*n* = 4; 33.3%), amoxicillin-clavulanic acid (*n* = 2; 16.7%) and ciprofloxacin (*n* = 1; 8.3%). *Pseudomonas aeruginosa* and the *Enterobacter cloacae* complex both showed resistance to the cephalosporins.

**TABLE 3:** Susceptibility profiles for specific bacterial isolates cultured from pus swabs of children with acute appendicitis.

Organism	Total (*N*)	Susceptible			Resistant			Intermediate		
		Agent	*n*	%	Agent	*n*	%	Agent	*n*	%
*Escherichia coli*	12	Trimethoprim-sulfamethoxazole	8	-	Trimethoprim-sulfamethoxazole	4	33.3	Cefuroxime (oral)	1	8.3
		Ampicillin and/or amoxicillin	7	58.3	Ampicillin and/or amoxicillin	5	41.7	-	-	-
		Nitrofurantoin	12	100.0	Amoxicillin-clavulanic acid	2	16.7	-	-	-
		Ciprofloxacin	11	92.7	Ciprofloxacin	1	8.3	-	-	-
		Cefuroxime (parenteral)	12	100.0	-	-	-	-	-	-
		Cefuroxime (oral)	11	92.7	-	-	-	-	-	-
		Cefoxitin	12	100.0	-	-	-	-	-	-
		Cefotaxime and/or ceftriaxone	12	100.0	-	-	-	-	-	-
		Ceftazidime	12	100.0	-	-	-	-	-	-
		Cefepime	12	100.0	-	-	-	-	-	-
		Gentamicin	12	100.0	-	-	-	-	-	-
		Amikacin	12	100.0	-	-	-	-	-	-
		Piperacillin-tazobactam	12	100.0	-	-	-	-	-	-
		Ertapenem	12	100.0	-	-	-	-	-	-
		Imipenem	12	100.0	-	-	-	-	-	-
		Meropenem	12	100.0	-	-	-	-	-	-
		Tigecycline	12	100.0	-	-	-	-	-	-
*Pseudomonas aeruginosa*	1	Ciprofloxacin	-	-	Trimethoprim-sulfamethoxazole	-	-	-	-	-
		Ceftazidime	-	-	Ampicillin and/or amoxicillin	-	-	-	-	-
		Cefepime	-	-	Nitrofurantoin	-	-	-	-	-
		Amikacin	-	-	Cefuroxime (parenteral)	-	-	-	-	-
		Piperacillin-tazobactam	-	-	Cefuroxime (oral)	-	-	-	-	-
		Imipenem	-	-	Cefoxitin	-	-	-	-	-
		Meropenem	-	-	Cefotaxime and/or ceftriaxone	-	-	-	-	-
		-	-	-	Tigecycline	-	-	-	-	-
*Enterobacter cloacae* complex	1	Trimethoprim-sulfamethoxazole	-	-	Amoxicillin-clavulanic acid	-	-	Nitrofurantoin	-	-
		Ciprofloxacin	-	-	Cefuroxime (parenteral)	-	-	-	-	-
		Cefepime	-	-	Cefuroxime (oral)	-	-	-	-	-
		Gentamicin	-	-	Cefoxitin	-	-	-	-	-
		Amikacin	-	-	Cefotaxime and/or ceftriaxone	-	-	-	-	-
		Ertapenem	-	-	Ceftazidime	-	-	-	-	-
		Imipenem	-	-	Piperacillin-tazobactam	-	-	-	-	-
		Meropenem	-	-	-	-	-	-	-	-
		Tigecycline	-	-	-	-	-	-	-	-

In six of the cases (42.9%), the bacteria showed resistance to ampicillin and/or amoxicillin, in five cases (35.7%) to trimethoprim-sulfamethoxazole and in three cases (21.4%) to amoxicillin-clavulanic acid. As shown in Table 2, no resistance was observed against cefepime, gentamicin, amikacin, ertapenem, imipenem, meropenem and colistin.

## Discussion

### Demographic characteristics

Our research showed that more cases of appendicitis occurred in males, with a male-to-female ratio of 1.5:1. This finding was consistent with several other South African studies on paediatric appendicitis, which consistently reported a male predominance.^22,26,27,28^ The male majority supports underlying biological and environmental determinants. The mean age in our study was 8 years, matching previous findings.^22,26,27,28^ This could indicate a constant age for paediatric appendicitis in South Africa.

### Microbial profile

Our research showed that there was no difference in species isolation between male and female patients. *Escherichia coli* was the most common organism, representing 85.7% of isolates. This was consistent with some studies (67% – 80%),^16,21,29,30^ but showed a higher occurrence than others (28% – 50%).^18,31,32,33^ *Escherichia coli* has been described as a notable cultured organism in appendicitis, indicating a diverse microbial profile in different groups.^34^ This variation could imply that while *E. coli* is frequently isolated in paediatric acute appendicitis, its prevalence can differ based on location and population characteristics. Therefore, it is essential to consider this organism when prescribing antibiotics.^35^ Understanding variations in antibiotic therapies can enhance treatment effectiveness, improve patient outcomes, and minimise complications from inappropriate treatment.

In our study, 30% *(n* = 6) of cases yielded no positive cultures, including four patients with complicated appendicitis (*n* = 4/6; 66.7%). These findings were consistent with recent literature, which reported a 39.2% rate of culture negativity in uncomplicated appendicitis cases.^36^ The absence of growth in some cases might be because of the nature of the infection (less severe cases) or the effectiveness of prior empiric antibiotic therapy, although not documented in this study.^21^ This highlights the complexity of appendicitis and the limitations of microbiological testing in predicting infectious outcomes.^36^ The findings underscore the importance of comprehensive clinical evaluation and using the most susceptible empiric antibiotic therapy for individual units to manage paediatric appendicitis effectively.

### Antibiotic sensitivity

All 14 organisms isolated from these patients were susceptible to cefepime, amikacin, imipenem, meropenem, ertapenem and gentamicin, including *E. coli*, the most common isolate.^16,18,29,30,32,33,37,38^ Other paediatric studies identified cefuroxime-metronidazole and ampicillin-sulbactam combinations^39^ as their most effective antibiotics.

Our study found an 85.7% sensitivity rate for cefotaxime-ceftriaxone, showing strong effectiveness, consistent with other studies.^18,32^ Combining ceftriaxone with metronidazole can be more effective than cefoxitin alone in treating uncomplicated appendicitis and reducing postoperative complications.^40,41^ Gentamicin had a 100% sensitivity rate, higher than the 68% – 82% range reported for aminoglycosides.^18,30,32,38^ However, recent paediatric studies do not mention gentamicin.^39,40^ The adverse effects associated with gentamicin, such as nephrotoxicity and ototoxicity, may limit its use.^42^ Clinicians should regularly monitor peak and trough levels when prescribing aminoglycosides to reduce the risk of adverse effects.^43^ Aminoglycosides are also ineffective against anaerobes.^44,45^ Therefore, adding metronidazole to the regimen in acute appendicitis cases is advisable,^40,41^ even though no anaerobes were isolated in this study with a small sample size.

In our study, susceptibility to cefuroxime was observed in 78.6% of the bacterial isolates for oral products, and 85.7% for parenteral preparations, aligning with broader trends in susceptibility to this cephalosporin observed in recent studies.^18,21^ Some Enterobacteriaceae produce ESBLs, conferring resistance to most β-lactam antibiotics, including new-generation cephalosporins. This may contribute to the cefuroxime sensitivity profile reported in the literature.^21^ It underscores the need for ongoing surveillance and adaptation of antibiotic regimens in clinical practice.

Our findings emphasise the necessity of customising antibiotic protocols based on local resistance patterns and clinical situations to improve outcomes. Continuous surveillance and region-specific research are essential for adjusting treatment protocols, especially given the global increase in antibiotic resistance.^17^ This approach enhances patient care and contributes to the global effort to promote antibiotic stewardship.

### Antibiotic resistance

We found 42.9% resistance rates for ampicillin-amoxicillin, 35.7% for trimethoprim-sulfamethoxazole and 21.4% for amoxicillin-clavulanic acid, making them fairly unreliable as first-line treatment for appendicitis in our unit. The rates for ampicillin-amoxicillin and trimethoprim-sulfamethoxazole resistance are higher than those reported in recent studies (15% – 32%),^16,39,40^ and reflect global concerns about increasing antibiotic resistance and the need for region-specific research to guide treatment.^17,30,33^ The findings highlight the urgent need to determine alternative therapies and improved stewardship programmes to address resistance trends.

Cefoxitin showed a 14.3% resistance rate, which was not mentioned in recent paediatric appendicitis studies.^18,39,40^ The resistance rate of 14.3% for cefotaxime and/or ceftriaxone was consistent with observed ranges for other antibiotics in paediatric appendicitis.^39,40^ Because of this resistance rate, we will not consider these two antibiotics as empiric treatment in our setting. This suggests the need to evaluate the effectiveness of different antibiotic agents in paediatric populations and use combination therapies to help overcome resistance and improve patient outcomes.

It is generally advised to avoid using antibiotics with resistance rates between 10% and 20%.^30^ Continuous monitoring of resistance patterns, adherence to guidelines and regular culture testing are crucial for tracking susceptibility, guiding antibiotic choices and ensuring effective treatments.

### Study limitations

The study has several limitations. Data were collected from a single tertiary public sector hospital, with a small sample size and short time period of the study, limiting its generalisability of the findings. The use of empiric antibiotic treatment was not documented from either referring institutions or our own pre-operative regimens potentially biasing our culture results. This study can serve as a pilot study to more extensive research in our unit. Despite these limitations, the results align with previous studies, and antibiotic treatment regimens can be adjusted for the unit.

### Recommendations

To optimise treatment outcomes, we recommend that empiric antibiotic treatment for acute appendicitis in our unit be changed to the local susceptibility patterns detected in this study. Intravenous ciprofloxacin and metronidazole can be used and then tailored to the susceptibility of the bacterial isolates based on the MCS results of the swabs taken intraoperatively. Given the high rate of susceptibility to ciprofloxacin, it can be used in empiric antibiotic therapy in acute appendicitis. Ciprofloxacin may cause adverse effects in 5% – 15% of paediatric patients,^46^ although these events (musculoskeletal effects, abnormal liver functions, nausea and vomiting, and changes in white cell counts) are usually reversible^47^ with no long-term sequelae. Cefepime also had a 100% susceptibility but will not be recommended for empiric treatment to preserve its effectiveness against *P. aeruginosa, Serratia* or *Enterobacter* infections in general. Future and ongoing regular culture testing should be conducted in all units to track susceptibility and guide antibiotic selection to ensure effective treatment of acute appendicitis in children.

### Rationale for the recommendations

Despite the 100% susceptibility in this study, using broad-spectrum antibiotics such as amikacin, imipenem, ertapenem and meropenem is discouraged for uncomplicated acute appendicitis because of stewardship concerns and potential drug resistance.^48^ The Surgical Infection Society (SIS) guidelines recommend reserving these drugs for high-risk or severe cases and providing education to improved appropriate antibiotic use without adverse outcomes.^48^ Simpler antibiotic regimens succeed in approximately 60% of uncomplicated cases, with outcomes comparable to surgery.^49,50^ Overusing potent antibiotics increases risks such as *Clostridium difficile* colitis, resistance, and major complications, reinforcing the importance of careful antibiotic selection.^49,51^

Metronidazole targets anaerobic bacteria such as *Bacteroides fragilis*, although not isolated in this study, possibly because of pre-operative antibiotic administration and the small sample size.^52,53,54^ It is cost-effective, can be given orally and reduces hospital costs by about 30% in complicated cases.^54^ It shortens hospital stays by about 1 day^53,54^ and helps prevent postoperative infections in severe cases.^55^ Its pharmacokinetics allow once-daily dosing.^56^

## Conclusion

*Escherichia coli* was the main bacterium cultured in acute appendicitis, followed by *P. aeruginosa* and the *E. cloacae* complex. Ciprofloxacin was 92.7% effective against *E. coli*. High resistance rates to ampicillin-amoxicillin, amoxicillin-clavulanic acid, and trimethoprim-sulfamethoxazole are unreliable as first-line treatments in our unit. Thus, ciprofloxacin and metronidazole are recommended for empiric management in our paediatric population. Metronidazole is recommended to cover potential mixed infections involving anaerobic bacteria, although no anaerobes were cultured in this study. Local resistance patterns should guide tailored antibiotic protocols to reduce broad-spectrum use and complications. Ongoing monitoring and region-specific research are essential for adapting treatment protocols and managing resistance. Further studies are needed to refine protocols, reduce costs and address evolving resistance patterns.
